# Detection of Flaws in Concrete Using Ultrasonic Tomography and Convolutional Neural Networks

**DOI:** 10.3390/ma13071557

**Published:** 2020-03-27

**Authors:** Marek Słoński, Krzysztof Schabowicz, Ewa Krawczyk

**Affiliations:** 1 Faculty of Civil Engineering, Cracow University of Technology, Warszawska 24, 31-155 Kraków, Poland; 2 Faculty of Civil Engineering, Wrocław University of Science and Technology, Wybrzeże Wyspiańskiego 27, 50-370 Wrocław, Poland; Krzysztof.Schabowicz@pwr.edu.pl (K.S.); krawczyk.em@gmail.com (E.K.)

**Keywords:** concrete, non-destructive testing, ultrasounds, ultrasonic tomography, acoustic methods, defects, diagnostic, detection, convolutional neural networks, transfer learning

## Abstract

Non-destructive testing of concrete for defects detection, using acoustic techniques, is currently performed mainly by human inspection of recorded images. The images consist of the inside of the examined elements obtained from testing devices such as the ultrasonic tomograph. However, such an automatic inspection is time-consuming, expensive, and prone to errors. To address some of these problems, this paper aims to evaluate a convolutional neural network (CNN) toward an automated detection of flaws in concrete elements using ultrasonic tomography. There are two main stages in the proposed methodology. In the first stage, an image of the inside of the examined structure is obtained and recorded by performing ultrasonic tomography-based testing. In the second stage, a convolutional neural network model is used for automatic detection of defects and flaws in the recorded image. In this work, a large and pre-trained CNN is used. It was fine-tuned on a small set of images collected during laboratory tests. Lastly, the prepared model was applied for detecting flaws. The obtained model has proven to be able to accurately detect defects in examined concrete elements. The presented approach for automatic detection of flaws is being developed with the potential to not only detect defects of one type but also to classify various types of defects in concrete elements.

## 1. Introduction

Concrete structural members need to be diagnosed at different times and for different reasons. For many years, attempts have been made to investigate geometrical and material imperfections in concrete members by exploiting the propagation of elastic waves. The current trend in the diagnosis of these elements is to apply a non-destructive testing equipment that allows one to obtain an image of the inside of the examined elements for early detection of flaws [1,2]. Acoustic techniques have seen greater attention because a clear shift has been toward acquiring more information about tested elements from acoustic signals [3,4,5,6,7,8]. Among the recently developed acoustic techniques, ultrasonic tomography (UT) stands out. Since it is still a relatively new approach, it has been used in a limited number of case studies. In Reference [9], it is recommended to identify defects in concrete members by means of the nondestructive ultrasonic tomography technique. The designers of the multi-probe measuring antenna, used in the ultrasonic tomograph, proposed to use ultrasonic tomography for the location of air voids in concrete members [10,11]. Samokrutov et al. [12,13,14,15] described the multiprobe antenna and its possible uses and presented an image-generating algorithm. On the basis of laboratory studies carried out using the tomograph, Schabowicz and Suvorov [16,17] introduced a change into the image-generating algorithm whereby it became possible to isolate the surface wave signals from the total picture of the wave and to remove noise for the benefit of the location of air voids in concrete members available from one side only. The other notable applications include practical use of ultrasonic tomography to test a unilaterally accessible concrete shell of heat pipe carrying tunnel [18] and to test non-destructive assessment of masonry pillars [19].

Currently, the recorded images of the inside of the examined concrete members, obtained from ultrasonic tomography, are inspected mainly manually, which can be expensive, time-consuming, and can be prone to errors. Moreover, there are no standards available, which could make it possible to objectively interpret the results obtained in this way. In that situation, a data-driven approach can be helpful because recent developments in computer hardware and software together with the corresponding advances in deep learning algorithms and availability of large datasets make fully automatic analysis of images possible [20,21,22,23,24,25]. Among deep learning algorithms, convolutional neural networks (CNNs) are currently the main research tools for automatic image analysis. CNNs go back to the 1980s, but, initially, they were mainly used for optical character recognition (OCR) [26,27]. At present, CNNs are commonly used for automatic extraction of information from data with a grid-like structure such as video and audio and for object detection, image classification, and image segmentation [28]. The use of CNN covers wide spectrum of applications such as in medical images analysis [29] and image-based civil infrastructure inspection and a condition assessment [30,31,32]. They are also applied in the context of ultrasound tomography for automatic analysis of ultrasound medical images [33,34]. On the other hand, the authors of this paper, are unaware of any applications of CNNs for automatic detection of flaws in concrete elements based on scans from ultrasonic tomography.

With the above in mind, this article contains a full description of the innovative methodology developed by the authors. The main objective of this work is, therefore, set to investigate the novel application of ultrasonic tomography and a convolutional neural network (CNN) for automating the assessment of defects and flaws in concrete elements. However, because it is the first attempt to tackle the flaws detection problem, this work involves few limitations. First, only one type of defect is considered. This assumes that the images with defects, obtained from ultrasonic tomography, belong to one category only. Second, in the experiments, only the images with easily visible flaws are considered. In future experiments, however, these two limitations will be considered to get a fully automatic system for detection and classification of defects in concrete elements.

The remainder of the paper is organized as follows. A brief description of ultrasonic tomography is presented. This is followed by a short introduction to artificial neural networks and a brief overview of convolutional neural networks. These provide the theoretical basis of the work undertaken in this paper. Next, we briefly discuss the proposed methodology for flaws detection. This is followed by a description of specific samples. The dataset is prepared, the neural network model is applied, numerical experiments are carried out, and the results of flaw detection are obtained. Lastly, a short discussion of the results and final conclusions are given.

## 2. Ultrasonic Tomography

Elastic wave stimulation in the tested element is called the ultrasonic tomography technique. Elastic wave stimulation in the tested element is called the ultrasonic tomography technique. The excitation source is a multi-head antenna. It contains several dozen integrated ultrasonic heads that generate 50 kHz ultrasonic pulses. This frequency is the most suitable for concrete and similar materials [11]. It is also used to receive and send ultrasonic signals with a maximum range not exceeding 2500 mm, which is the most reliable range for the ultrasonic tomographs currently in use [11]. The validity of using ultrasonic tomography for concrete elements results from the fact that concrete has a highly heterogenite inner structure, which causes a high level of structural noise and a fundamental ultrasonic signal damping during ultrasonic testing [11].

**Figure 1:**
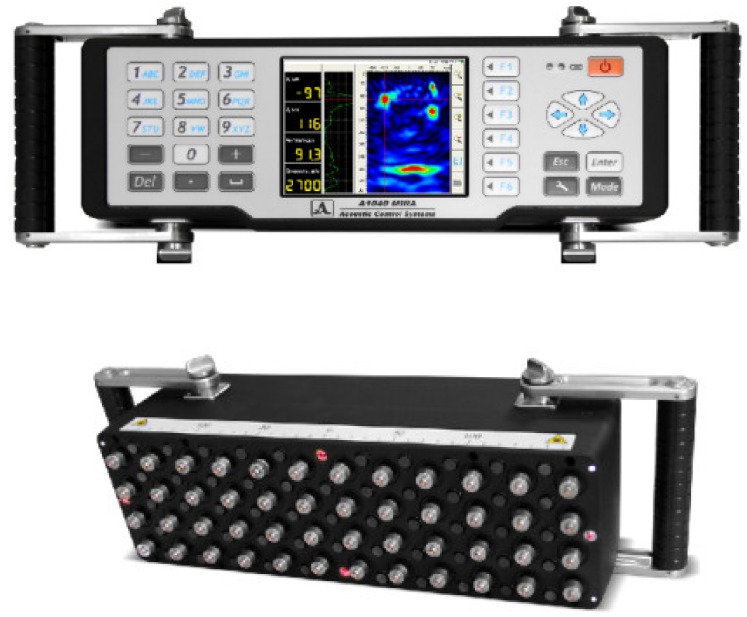
Ultrasonic tomograph: top and bottom view.

Figure 1 shows the new ultrasonic tomograph. The tomograph includes a multi-head ultrasonic antenna with a computer and a customized software suitable for graphic scan recording.

**Figure 2:**
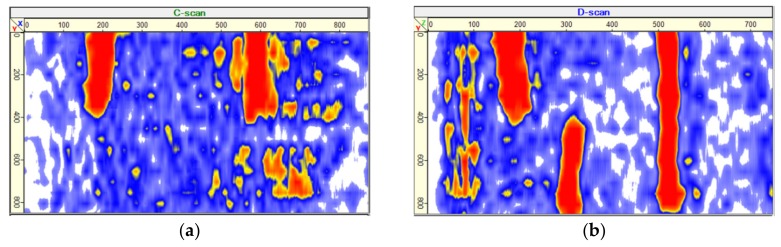

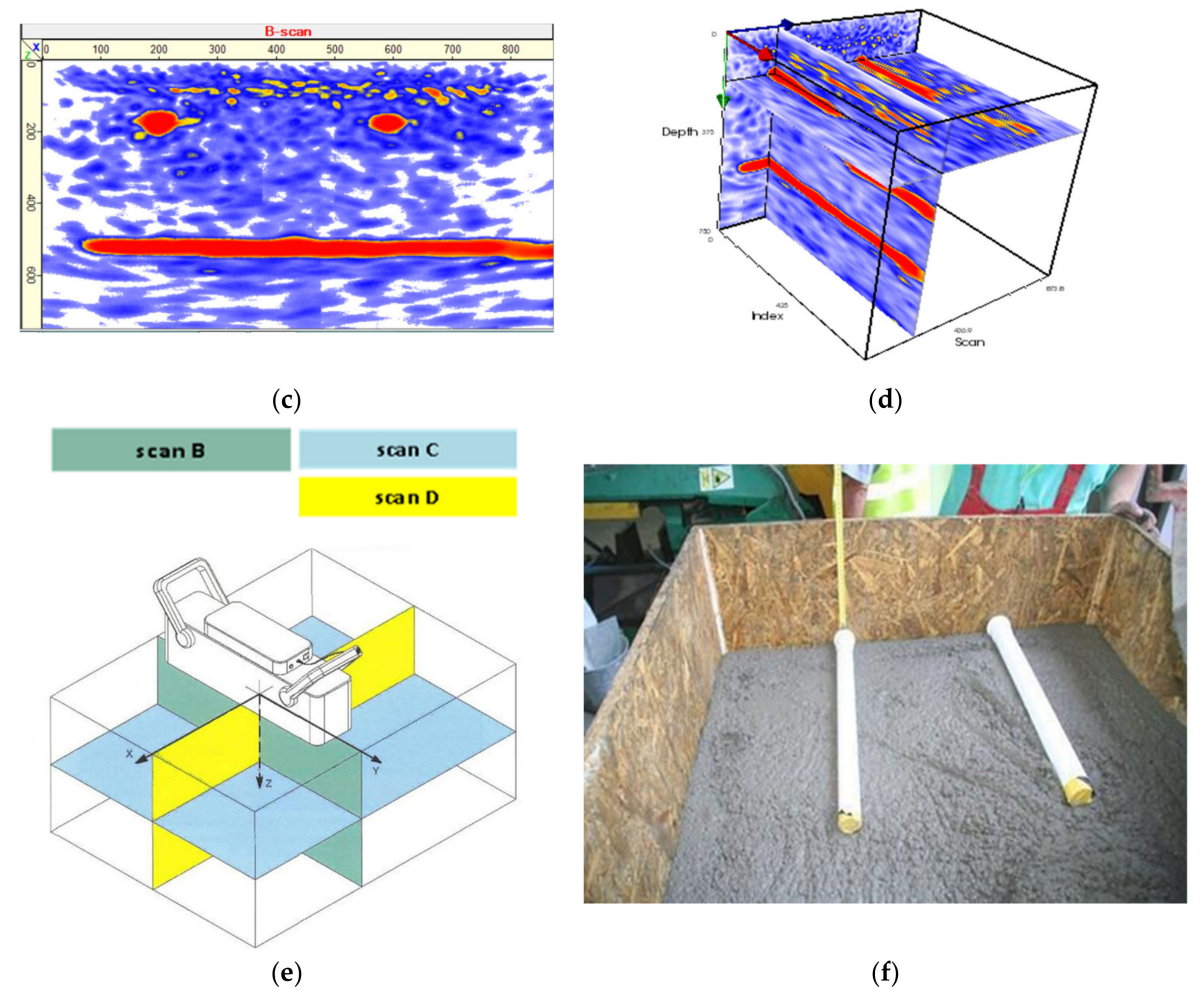
Example of ultrasonic tomography scans for a concrete member filled with air PVC pipes: (**a**) exemplary scan C, (**b**) exemplary scan D, (**c**) exemplary scan B, (**d**) 3D scan, (**e**) names of cross sections (scans) of tested object and coordinate system used with a tomograph antenna, and (**f**) the view of the embedded PVC.

Each head is telescopically mounted separately in the antenna, and the head adapts to the testing surface. Thanks to dry contact, no coupling agents or special surface preparation for testing is required. The distance between the heads is 30 mm and 40 mm, respectively, both vertically and horizontally. Figure 2 shows the results of ultrasonic tomography tests in the form of C, D, and B scans and a 3D scan for a concrete element with a defect modeled by PVC (Poly Vinyl Chloride) pipes filled with air because ultrasonic tomography is more sensitive to air voids than to PVC pipes filled with air. The diameter of PVC pipes was 25 and 35 mm.

The results of testing using the ultrasonic tomography are then collected in a matrix table. This matrix is three-dimensional and is subsequently processed by the special software. In that way, it is possible to receive three scans: C, D, B, and 3D view in the mutually perpendicular directions, as shown in Figure 2d. Figure 2e shows names of three mutually perpendicular cross sections (scans) of the tested object and coordinate system used with tomograph antenna.

## 3. Convolutional Neural Networks

The convolutional neural network (CNN) is a special type of a layered feed-forward artificial neural network (ANN), designed for processing signals in the form of multiple arrays like visual and audio signals [21]. The standard layered feed-forward neural network architecture is called a multi-layer perceptron (MLP) [35]. It has been used for many years but has several shortcomings. Up to the mid-2000s, the most popular neural network architecture had one or two hidden layers (i.e., shallow network). If the network has more than two hidden layers, the network is called a deep neural network (DNN).

The typical neural network like MLP or CNN has an input layer, at least one hidden layer with nonlinear units (neurons), and an output layer with linear units (for regression problems) or nonlinear units (for classification problems). A unit (neuron) computes a weighted sum of its inputs called activation of the unit and then the activation is sent to the activation function, which is, in general, an S-shaped function such as a sigmoid function or a rectified linear unit (ReLU) function.

The number of inputs in the input layer is equal to the total number of features in the input dataset. For example, for RGB images (three channels) with 150 × 150 pixels for each channel, the number of inputs is 67,500. The number of outputs depends on the problem at hand. For example, for the binary classification problem, there is only one output. For multi-class classification, the number of outputs corresponds to the number of classes.

**Figure 3:**
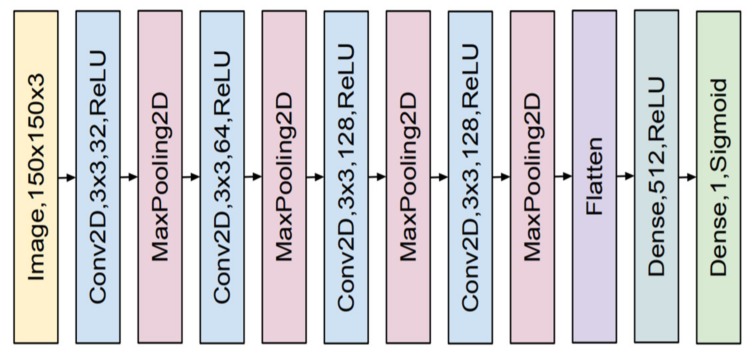
Example of the convolutional neural network (CNN) structure for processing the RGB image of 150 × 150 pixels in size. The CNN model consists of four convolutional layers with various number of the 3 × 3 kernel and the ReLU activation function (blue rectangles) with each followed by a pooling layer (red rectangles) and two fully-connected (dense) layers (green rectangles) after a flattened operation.

A CNN has a slightly different architecture. It consists mainly of two types of hidden layers, which are a convolutional layer and a pooling layer. There is also a fully-connected (dense) layer that forms the network outputs. These layers are stacked to form a convolutional neural network structure. The structure of a typical CNN with 11 layers is presented in Figure 3.

The aim of the convolutional layer is to detect the input image local features such as horizontal, vertical, or diagonal edges. It is done by using a convolution operation or by computing cross-correlation more strictly [21].

The pooling layer is used for dimensionality reduction of the processed data by combining values of small clusters (matrices) of input data. The most common pooling operation is defined by taking the maximal value. Additionally, the flattened operation is needed to change the input matrix to the output vector, which was then processed by the fully-connected (dense) layer. The fully-connected layer processes the input data by sending data from every unit (neuron) in the previous layer to every unit in another layer.

A layered neural network is qualified by using the backpropagation algorithm to efficiently compute the gradient of a loss function and the mini-batch stochastic gradient descent algorithm (SGD) for learning the weights of the neural network model. During training of convolutional neural networks with many parameters (several millions), especially on a small dataset, the main issue is overfitting the model to the training dataset. There are several methods and techniques to cope with overfitting [36].

One of the most effective techniques is image data augmentation [37]. This technique allows us to expand the training dataset via a number of random transformations such as rotation, shift, zoom, and flipping, which produce visually similar images. Another regularization technique is the dropout method, which is a technique to improve training process performance by switching off (setting to zero) some number (usually 50%) of randomly selected weights [38].

Another problem with learning the large convolutional neural network is the extra-long time needed for a successful training process. One of the possible solutions is to apply transfer learning. Transfer learning is a method used to adapt the pre-trained model to another dataset [39]. It is often done with fine-tuning, which is a technique to improve the performance of the neural network by adapting selected weights and by applying additional training to several convolutional layers of CNN while the rest of the layers are preserved. 

There are many commonly used pre-trained CNN models (for example, AlexNet, VGG-16, U-Net), which can be used in other problems by applying transfer learning. The pre-trained network has parameters that were computed on a very large training dataset from a specific domain or task. For example, the benchmark dataset called ImageNet is used for testing novel CNN structures and training algorithms [40].

## 4. Experimental Study

### 4.1. Testing Methodology

**Figure 4:**
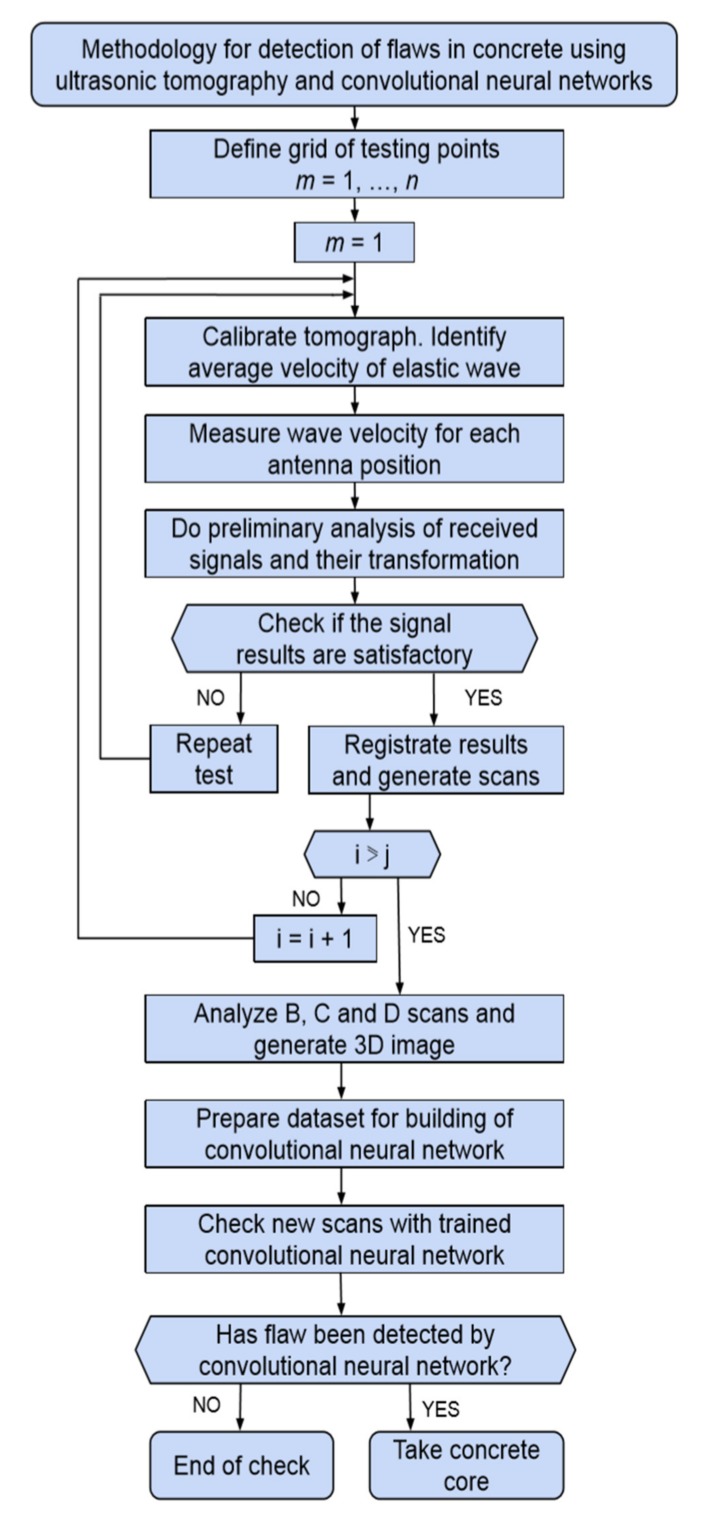
Flowchart presenting methodology for detecting flaws in concrete elements using ultrasonic tomography and convolutional neural networks.

The methodology for detecting flaws in concrete elements using ultrasonic tomography and convolutional neural networks is shown in the form of a flowchart in Figure 4 and is described in detail below.

The first step in the testing methodology consists of marking a grid of measuring points i = 1…j evenly spaced at every 50 mm, with a minimum distance of 50 mm from the edge of the tested concrete member. The spacing can be increased to 100 mm if the surface of the investigated member is considerable. The tomograph is then calibrated by repeatedly measuring ultrasonic wave (signal) velocity and computing its mean value.

At subsequent steps, ultrasonic wave velocity is measured in each antenna position in each of the testing points. During measurements, a preliminary analysis of the ultrasonic signals is performed to find out if the thickness of the member can be identified or the defect can be detected on this basis. If this is not the case, the measured signals are transformed using the customized software. The transformation consists of compiling the registered data for a given measuring point.

If the results are acceptable, they are recorded. Lastly, flat scans B, C, and D are obtained in the three mutually perpendicular directions, showing the inside of the investigated concrete member. By using the dedicated software, it is also possible to build a three-dimensional scan. The next step is analyzing scans B, C, and D, and forming a set of data used for building (training and testing) the convolutional neural network (CNN). Lastly, the trained CNN is presented with a new scan and the network analyzes the new scan and decides if the scan contains a flaw or not.

The presented methodology of measuring and processing obtained results using ultrasonic tomography and convolutional neural networks can be useful when developing a prototype of a computer vision system to automatically detect flaws in concrete elements.

### 4.2. Dataset for Building Convolutional Neural Networks

For the purpose of this work, ten 1000 × 1000 × 1000 mm^3^ concrete cubic specimens were prepared. The specimens were made of C25/30 concrete based on aggregates with 8-mm maximum grading. Next, the specimens were tested in the laboratory using the technique of ultrasonic tomography and the images containing B-scans were recorded. The images were cropped to prepare a dataset, which was used to train a convolutional neural network. A CNN-based detection model was built using a dataset containing only 246 B-scans of concrete elements with flaws (52%) and without flaws (48%). The dataset was divided into three subsets: for training (56%), for validation (22%), and for testing (22%). Table 1 contains the number of training, validation, and testing samples, respectively.

**Figure 5:**
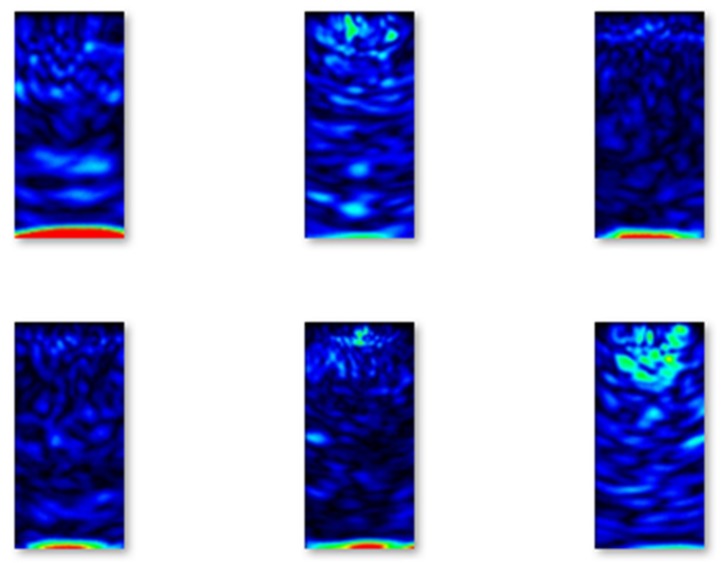
Selected examples of B-scans without flaws from the training set.

In Figure 5, images of B-scans of concrete elements without flaws from the training set are shown.

**Figure 6:**
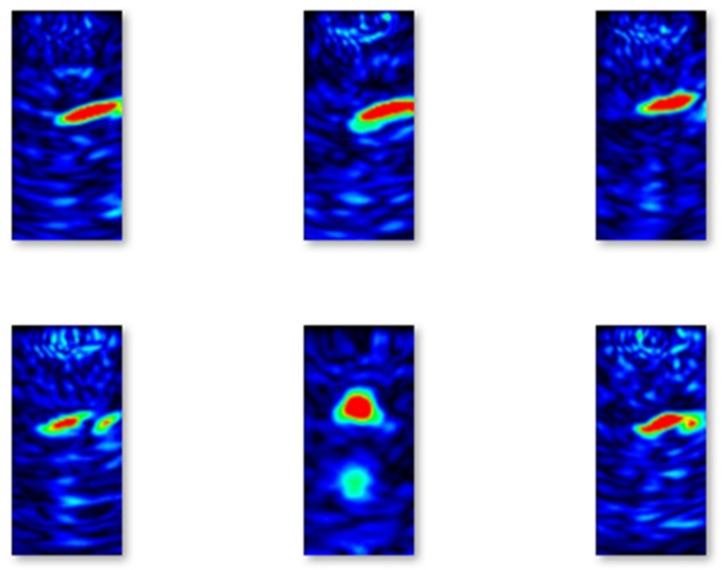
Selected examples of B-scans with flaws from the training set.

Similarly, in Figure 6, images of B-scans of concrete elements with flaws from the training set are shown. Comparing the images, it can be easily seen that the images taken from concrete elements with flaws contain red oval-shaped parts that correspond to the location of the flaws.

**Figure 7:**
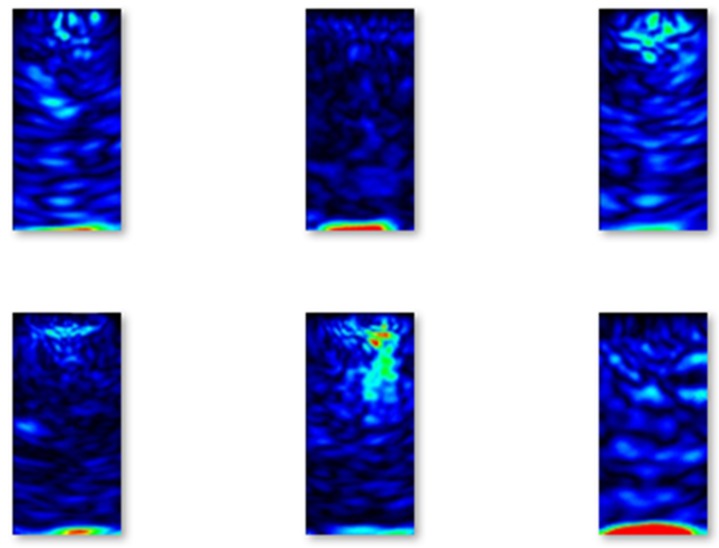
Examples of B-scans without flaws from the validation set.

Selected examples of B-scans without flaws from the validation set are shown in Figure 7.

**Figure 8:**
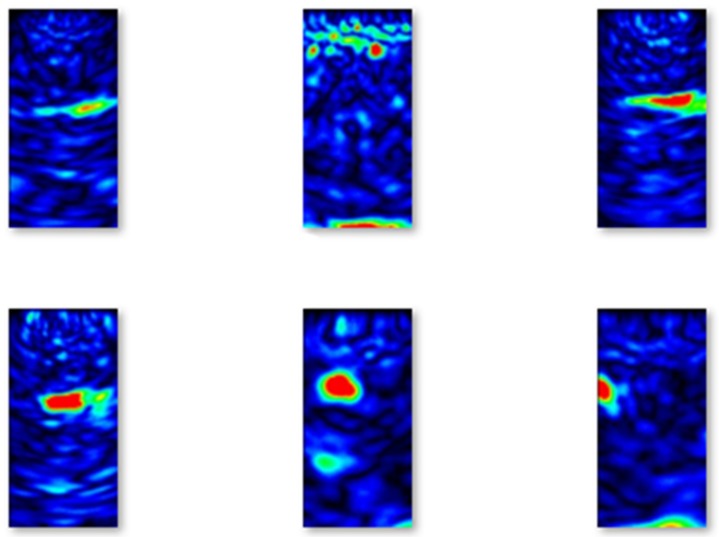
Examples of B-scans with flaws from the validation set.

Selected examples of B-scans with flaws from the validation set are shown in Figure 8.

**Figure 9:**
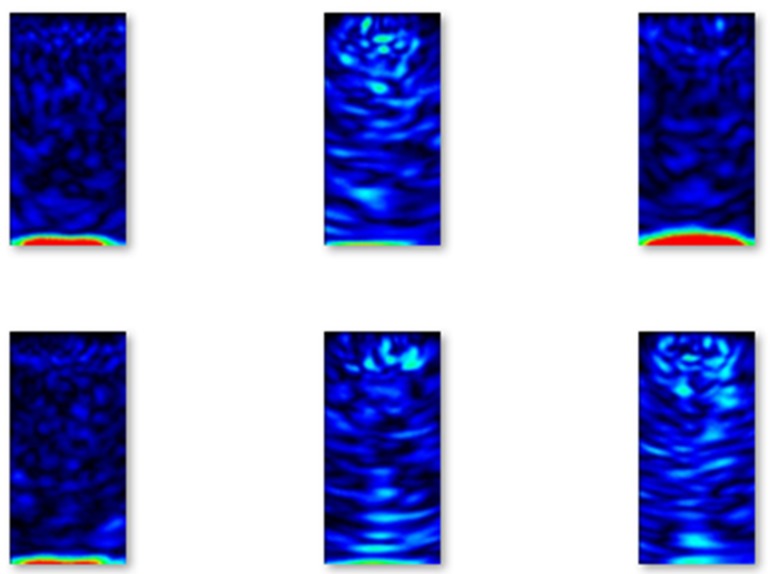
Examples of B-scans without flaws from the testing set.

Selected examples of B-scans without flaws from the testing set are shown in Figure 9.

**Figure 10:**
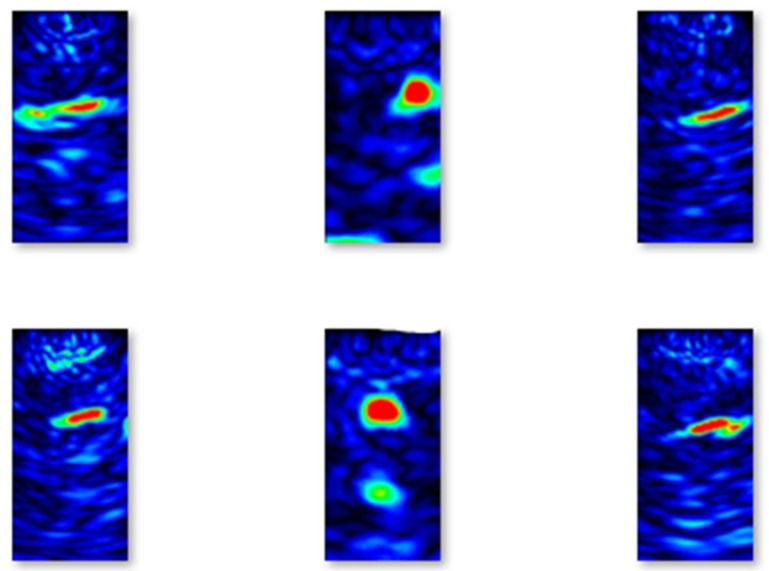
Examples of B-scans with flaws from the testing set.

Lastly, selected examples of B-scans with flaws from the testing set are shown in Figure 10.

### 4.3. Experimental Setup

**Figure 11:**
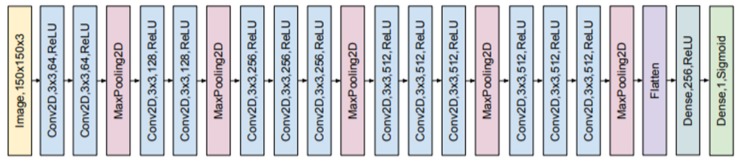
Structure of a pre-trained convolutional neural network with 21 layers (VGG-16).

In this work, to achieve better results in automatic flaw detection, we adopted transfer learning and used a pre-trained convolutional neural network called VGG-16. It was proposed in 2014 by K. Simonyan and A. Zisserman from the Visual Geometry Group at the University of Oxford [41]. VGG-16 was trained on more than a million images from the ImageNet database [40]. This database contains over 14 million images belonging to 1000 classes and was designed for the development of new deep learning algorithms for visual object recognition. VGG-16 was the winner of the ImageNet Challenge in 2014. The network consists of 21 layers including 13 convolutional layers with a filter size of 3 × 3. The VGG-16 architecture is shown schematically in Figure 11.

We use the convolutional base of the pretrained network trained with an image augmentation binary classifier set on the top of the convolutional base. We also apply fine-tuning, introduced in Section 3, for training weights in the last three convolutional layers to improve the performance of the classifier. Table 2 contains the basic parameters such as the number of layers, the total number of parameters, and the size of the considered network.

The input for the network was the normalized image of the CT scan. The input image was then processed by several convolutional and max pooling layers. The kernels in the convolutional layers have the same size of 3 × 3 and a different number of filters. All convolutional layers used the ReLU activation function. Max pooling layers used a stride of size 2. After all convolutional and max pooling layers, the flattening operation is applied and then the resulting vector of features is processed by a fully connected layer with a ReLU function. Lastly, the input is classified by the last layer, which is fully connected and has the sigmoid function. The convolutional neural network was trained by using the Adam optimizer to optimize the cross-entropy loss function.

The training process includes only fine-tuning of the last block of convolutional layers during 100 epochs of regularized training with image augmentation and dropout (50%). The CNN model is trained, validated, and tested by applying the corresponding datasets presented in Section 4.2.

The numerical experiments were prepared in the Python ecosystem using Keras library [42]. The computations were performed on a Dell Inspiron 15 laptop computer with 64-bit Windows 10, 32 GB RAM memory, Quad-Core Intel Core i7 processor, and NVIDIA GeForce GTX 1060 Ti (4 GB) graphics processing unit (GPU).

## 5. Results

In this section, the training process and the results of the experiments as well as the final performance of the convolutional neural network (CNN) for image-based detection of flaws in concrete elements are presented.

**Figure 12:**
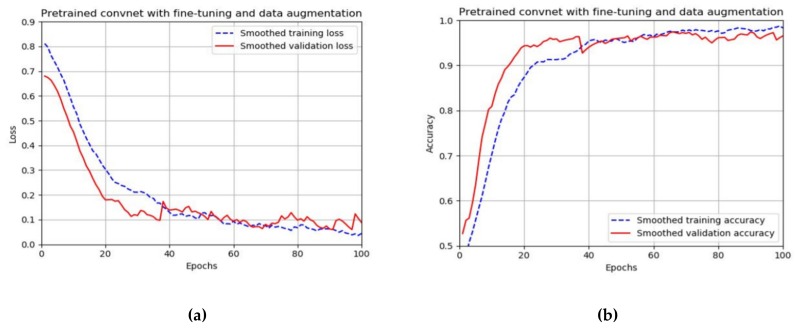
Performance of convolutional neural network (CNN): (**a**) training and validation losses, (**b**) training and validation accuracies.

In Figure 12, the training and validation losses for the network applied in the experiments are presented. From the plots, it can be observed that the minimal training loss achieved for the network with image augmentation and fine-tuning are 0.04 and 0.06. In Table 3, the lowest training and validation losses and training time for one epoch are given.

In Figure 12, the training and validation accuracies during training within 100 epochs are presented. It can be seen that the best results obtained in terms of accuracy are similar to the corresponding results in terms of loss. The training accuracy after 100 epochs is 98% while the maximal validation accuracy is 97%. Table 4 shows the highest training and validation accuracies. The table also contains the times needed to perform one epoch of training. The training time for the large pre-trained CNN with image augmentation and fine-tuning is 18 s per epoch.

**Figure 13:**
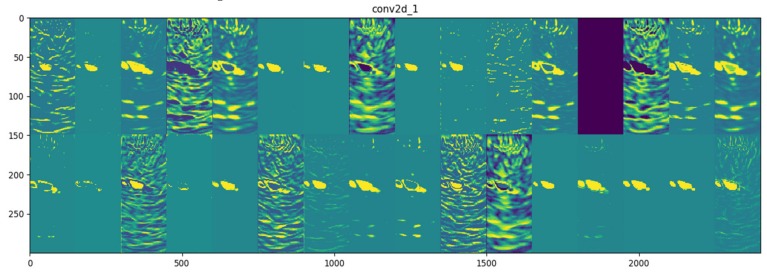
Visualization of 2 × 16 filters from the first convolutional layer.

After the training process, it is possible to visualize the filters of the convolutional and pooling layers. It can be useful to better understand how the CNN model represents the visual information from the training dataset. In Figure 13, the image shows the visualization of 32 filters from the first convolutional layer, which is shown in the form of merged sub-images (2 rows and 16 columns). The size of each filter is 150 × 150 px.

**Figure 14:**
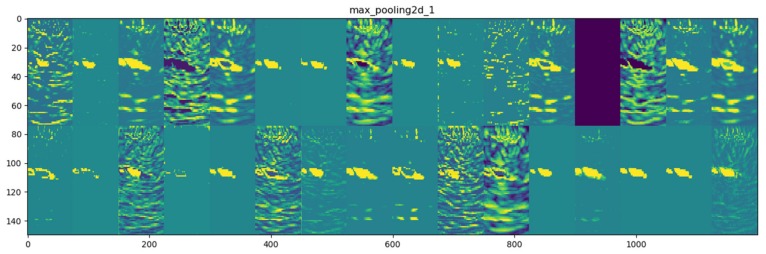
Visualization of 2 × 16 filters after the first max pooling layer.

In Figure 14, the image shows the visualization of the corresponding 32 filters after the first pooling layer. The size of each filter is 75 × 75 px.

Lastly, the trained convolutional neural network was checked against generalization capacity by using the testing dataset. After analysing the new B-scans by the network after fine-tuning, the generalization accuracy for the final network was close to 99%.

## 6. Discussion

The presented work is concerned with the development of novel methodology for fully automated analysis of images of the inside of the examined concrete elements obtained from an ultrasonic tomograph. The proposed methodology is based on the combination of the ultrasound tomography technique and the convolutional neural network (CNN). The CNN model is applied at the final stage of the developed methodology for classifying images into two categories: “element without a flaw” and “element with a flaw.”

Because the main challenge was to collect enough data, instead of building the CNN model from scratch, we applied a pre-trained convolutional neural network called VGG-16 trained on the ImageNet dataset. The parameters of the pre-trained model were fine-tuned by using the collected images and the transfer learning technique. Moreover, two regularization techniques were applied to avoid problems with overfitting and obtain a satisfactory generalization property of the final CNN model. The first technique was image data augmentation by using basic transformations of an image-like rotation, horizontal flipping, and shifts. The second technique was network weights pruning called dropout. In the process of building the neural model, only 246 RGB images were used. After 100 epochs of training, the network achieved 98% accuracy on the training set with a loss of 0.05. The validation accuracy was 97% with a loss of 0.09. Lastly, the generalization accuracy was close to 99%, which confirms that the CNN model was built properly.

Analysis of the obtained results shows that it is very possible to detect defects in concrete samples with very high accuracy. In the provided example, the concrete cubic laboratory specimens were tested using ultrasonic tomography and the trained convolutional neural network. The experiments were carried out according to the proposed testing approach.

## 7. Conclusions

The innovative methodology of non-destructive detection of defects in concrete elements using ultrasound tomography and a convolutional neural network has been presented in detail. There are two main stages for detecting defects in concrete elements, according to the presented methodology. The first of these is to carry out non-destructive testing using ultrasound tomography techniques. The second stage is automatic defect detection using a convolutional neural network. Interesting results on building practices are given in the provided case study.

- A practical example of the application of the proposed methodology was presented,
- By using two regularization techniques and transfer learning, we were able to significantly reduce the risk of overfitting the model during training,
- In the end, we obtained the CNN-based classifier, which is able to generalize well on unseen images,
- The accuracy of detecting flaws using convolutional neural networks has been confirmed,
- The usefulness of the presented methodology for non-destructive identification of defects in concrete elements was shown,
- The proposed solution can serve as a prototype in the context of future practical applications.

The benefit of the proposed methodology is based on the fact that the combination of ultrasonic tomography and convolutional neural networks offer a method to build a computerized system for complete automatic detection of flaws in concrete elements when given a very small number of recorded image datasets. Based on this research experience, the authors have found that the developed methodology has practical applications in future automatic inspection systems.

In the future, because this was the first attempt to tackle the problem of automatic flaws detection, the limitation and challenges that were faced in this work will be addressed. First, we considered only one type of defect simulated by a PVC pipe embedded in the concrete element. As a result, the images with such a defect belong to one category only. Second, only the images with easily visible flaws were taken into account. In future work, the presented limitations will be considered by testing concrete specimens with other defects to be able to get closer to an automatic system for detection and classification of flaws in concrete elements.

## Figures and Tables

**Table 1:** Number of training, validation, and testing samples.

Dataset	With Flaws	Without Flaws	Total
Training	71	66	137
Validation	29	26	55
Testing	28	26	54
Total	128	118	246

**Table 2:** Basic parameters of the applied network.

CNN Model	#Convolutional Layers	#Layers	#Parameters
VGG-16	13	21	16,812,353

**Table 3:** Training and validation losses and training time for one epoch.

Convolutional Neural Network (CNN) Model	Training Loss	Validation Loss	Time (s)
VGG-16	0.04	0.06	18

**Table 4:** Training and validation accuracies and training time for one epoch.

CNN Model	Training Accuracy	Validation Accuracy	Time (s)
VGG-16	98%	97%	18
